# Human time perspective and its structural associations with voxel-based morphometry and gyrification

**DOI:** 10.1007/s11682-020-00416-1

**Published:** 2020-12-03

**Authors:** Simon Schmitt, Bianca Besteher, Christian Gaser, Igor Nenadić

**Affiliations:** 1 Department of Psychiatry and Psychotherapy, Philipps-University Marburg / Marburg University Hospital - UKGM, Rudolf-Bultmann-Str. 8, 35039 Marburg, Germany; 2 Department of Psychiatry and Psychotherapy, Jena University Hospital, Jena, Germany; 3 Department of Neurology, Jena University Hospital, Jena, Germany

**Keywords:** Magnetic resonance imaging (MRI), Time perspective, Voxel-based morphometry (VBM), Gyrification, Zimbardo Time Perspective Inventory (ZTPI)

## Abstract

Time perspective refers to humans’ concept of integrating and evaluating temporal position and evaluation of memories, emotions, and experiences. We tested the hypothesis that different aspects of time perspective, as assessed with the Zimbardo Time Perspective Inventory (ZTPI) are related to variation of brain structure in non-clinical subjects. Analysing data from n = 177 psychiatrically healthy subjects using voxel-based morphometry with the CAT12 software package, we identified several significant (p < 0.05 FWE, cluster-level corrected) associations. The factors past negative, reflecting a negative attitude towards past events and present fatalistic, measuring a hopeless and fatalistic attitude towards future life, were both negatively associated with grey matter volumes of the anterior insula. The ZTPI factor future was negatively associated with precuneus grey matter. There was no association of ZTPI scores with gyrification using an absolute mean curvature method, a marker of early brain development. These findings provide a link between a general psychological construct of time perspective and brain structural variations in key areas related to time keeping (anterior insula) and the default mode network (precuneus), both of which overlap with variation in behavioral aspects and psychopathology.

## Introduction

The concept of time perspective in psychology refers to “cognitive frames” of temporal processing, which include macro-level “encoding, storing, and recalling experienced events”, as well as the formation “of expectations, goals, contingencies, and imaginative scenarios” (Zimbardo and Boyd 1999). In the broader concept of processing of time on the neural level (Wittmann 2009, 2013), temporal perspective goes beyond simple estimated comparisons of temporal features. Rather, in the conceptualisation put forward by Zimbardo and Boyd (1999), time perspective is thought to reflect a fundamental and pervasive (although often not conscious) factor in judgments, decision making and initiation of actions (Rönnlund et al. 2019). In order to quantify deviations from optimal time perspective profiles the construct balanced time perspective has been introduced which refers to the mental ability to switch flexibly among time perspective in order to adapt to certain circumstances (Stolarski et al. 2011; Zimbardo and Boyd 1999).

The development and validation of the Zimbardo Time Perspective Inventory (ZTPI) has enabled the use of a self-report questionnaire capturing essential aspects of interindividual differences in time perspective, and its use in a range of applications spanning personality psychology, clinical psychology/psychopathology (Adams and Nettle 2009; van Beek et al. 2011; Zimbardo and Boyd 1999), and more recently also imaging studies (Carelli and Olsson 2015; Guo et al. 2017; Wittmann 2013; Wittmann et al. 2011).

The ZTPI shows five basic factors for time perspective (Zimbardo and Boyd 1999): (1) Past negative, which “reflects a generally negative, aversive view of the past” possibly linked to “unpleasant or traumatic events” (e.g. “I often think of what I should have done differently in my life”), (2) Present hedonistic, which “reflects a hedonistic, risk-taking, ‘devil may care’ attitude toward time and life” and has been assumed to suggest “an orientation toward present pleasure with little concern for future consequences” (e.g. “I try to live my life as fully as possible, one day at a time”), (3) Future: describing “behavior (that) is dominated by a striving for future goals and rewards” and a “general future orientation” (e.g. “Before making a decision, I weigh the costs against the benefits”), (4) Past positive, a factor which “reflects a warm, sentimental attitude toward the past” (e.g. “I keep working at difficult, uninteresting tasks if they will help me get ahead”), and finally (5) Present fatalistic: a “fatalistic, helpless, and hopeless attitude toward the future and life” (e.g. “My life path is controlled by forces I cannot influence”).

Time perspective is related to a range of cognitive processes as well as resilience and psychopathology (Blomgren et al. 2016; Zhang et al. 2013). For example, it is associated with interindividual differences in impulsivity (Baumann and Odum 2012; Wittmann et al. 2011) and personality (van Beek et al. 2011), as well as prediction of drug use (Apostolidis et al. 2006), perceived stress (Rönnlund et al. 2018), ageing (Laureiro-Martinez et al. 2017), chronotype (Stolarski et al. 2013), well-being (Cunningham et al. 2015; Garcia et al. 2016; Tseferidi et al. 2017) and addictive behaviors (Cheong et al. 2014; Fieulaine and Martinez 2010; Hodgins and Engel 2002; Keough et al. 1999; Kim et al. 2017; Pluck et al. 2008). Studies of psychiatric patients or symptoms have shown multiple correlations with aspects of time perspective, including disorders like depression (Garcia et al. 2016; McKay et al. 2017; Stolarski and Matthews 2016), anxiety disorders (Åström et al. 2018) and attention-deficit hyperactivity disorder (Carelli and Wiberg 2012), as well as suicidal behaviors (Laghi, Baiocco et al. 2009). This suggests that while capturing important aspects of psychopathology, time perspective might be of trans-diagnostic relevance, i.e. a general factor permeating diagnostic boundaries with relevance for a wide range of functional as well as dysfunctional behaviors.

Furthermore, in the non-clinical spectrum, different aspects of time perception have been linked to well-being, health behaviour, loneliness and feeling depressed (Bergman and Segel-Karpas 2018; Drake et al. 2008). Imaging studies also showed an increase in grey matter volume in the right precuneus associated with subjective happiness in healthy participants (Sato et al. 2015).

Recently, functional as well as structural neuroimaging studies started to investigate neuronal correlates of time perspective (Carelli and Olsson 2015; Chen et al. 2018). While these study provide initial leads of links between time perspective and its functional and structural representation in the brain, it leaves open the question whether time perspective is also related to other brain structural markers e.g. gyrification.

Individual time perspective is also playing a crucial role when investigating neural correlates of time perception (Carelli and Olsson 2015). Using functional MRI, the future perspective factor of the ZTPI was linked to brain activation during a duration reproduction task (time intervals ranged from 3 to 18 seconds) in a “core control network” including the insula, inferior and medial frontal cortices and inferior parietal cortices (Wittmann et al. 2011). An important conclusion of these findings was that this core control network may form a biological marker for cognitive time management.

Human’s cognitive ability to process longer time ranges has been investigated in time travel studies, in which participants are asked to mentally envision themselves in different temporal contexts that can either be in the past or future. Independent from whether participants thought about past or future contexts, brain areas were activated (comprising of the medial prefrontal cortex, parietal cortex, medial parietal cortex and medial temporal lobes; Addis et al. 2007; Botzung et al. 2008; Szpunar et al. 2007), overlapping with areas associated with episodic memory. This suggests that similar neural and cognitive processes are subject to episodic memory retrieval as well as the construction of events during mental time traveling. Another study (Abraham et al. 2008), in contrast, did not show activation in areas like the precuneus (involved in autobiographical memory retrieval) when participants engaged in mental time travel without imagining themselves in the past or future scenario. Yet, it remains unclear, whether time traveling is directly related to time perspective, as suggested previously (Carelli and Olsson 2015).

In the present study, we tested the hypothesis that interindividual differences in time perspective (as assessed by ZTPI) are associated with brain structural variation, in particular grey matter volumes and gyrification.

## Methods

### Subjects

Our study sample included 177 psychiatrically healthy volunteers (82 male, 94 female; mean (age) = 29.86, SD (age) = 8.93, median (age) = 26.44), who gave written informed consent to a study protocol approved by the Ethics Committee of the Medical School of Friedrich-Schiller-University of Jena. Prior to inclusion into the study, all subjects were carefully screened to exclude current or previous psychiatric disorders, including substance dependence, as well as central nervous pathologies/central neurological conditions, traumatic brain injury or uncontrolled major medical conditions such as hypertension or diabetes. Participants were also screened to exclude subjects with a first-degree relative with a psychotic disorder. All subjects had an estimated IQ > 80 (mean (IQ) = 106, SD (IQ) = 11.5, range (IQ) = 86–143), as assessed with the MWT-B (Mehrfachwortschatztest-B), a German equivalent of the National Adult Reading Test (NART). We also assessed handedness with the Edinburgh Handedness Inventory (Oldfield 1971): mean (EHI) = 80.53, SD (EHI) = 20.6. At the time of study, all subjects completed the Zimbardo Time Perspective Inventory (Zimbardo and Boyd 1999), using a validated German version (Funke et al. 2009).

### Magnetic resonance imaging (MRI) acquisition

We obtained high-resolution structural MRI scans on a 3 T Siemens Tim Trio system (Siemens, Erlangen, Germany) using a standard quadrature head coil and MP-RAGE sequence with 192 contiguous sagittal slices (TR = 2300 ms, TE = 3.03 ms, flip angle 9°, in-plane field-of-view 256 mm, voxel resolution 1 × 1 × 1 mm; acquisition duration 5:21 min). Scans were manually checked for absence of artefacts precluding further processing.

### Voxel-based morphometry (VBM) and gyrification analysis

For VBM analysis, we used the CAT12 toolbox (www.neuro.uni-jena.de/cat; Gaser et al. in review). CAT12 is a toolbox implemented in the SPM12 software package (Penny et al. 2011). As detailed in a recent study (Besteher et al. 2017), scans were corrected for bias – field inhomogeneities, then spatially normalized using the DARTEL algorithm (Ashburner 2007) and segmented into grey matter, white matter and cerebrospinal fluid (CSF`; Ashburner and Friston 2005). In the segmentation process we accounted for partial volume effects (Tohka et al. 2004), applying adaptive maximum a posteriori estimations (Rajapakse et al. 1997) and using a hidden Markov Random Field model (Cuadra et al. 2005). For exclusion of artefacts on the grey–white matter border (i.e. incorrect voxel classification), we applied an absolute grey matter threshold of 0.1. After pre-processing all scans underwent (and passed) automated quality control implemented in CAT12. Modulated grey matter intercorrelations ranged from 0.82 to 0.92, which indicates high sample homogeneity. For smoothing, we used a smoothing kernel of 12 mm (FWHM). Cluster labelling was conducted by using the neuromorphometrics atlas for DARTEL space.

Using CAT12 default settings, we further extracted cortical surfaces (Dahnke 2013), from which we computed local gyrification values for each participant based on absolute mean curvature (Luders et al. 2006). These datasets were then smoothed with a Gaussian kernel of 15 mm full width at half maximum (FWHM).

### Statistics

For statistical analysis of the VBM data in SPM12, we used separate multiple regressions for each ZTPI variable, which we used as regressor. Age, sex and total intracranial volume (TIV) were added to our model as covariates to remove any variance related to them. For each ZTPI scale, we analyzed positive and negative associations with grey matter volume by first applying a peak threshold of α = 0.001, followed by cluster-level corrected levels at *p* < 0.05 with FWE-correction. We also described the sizes of the found clusters by mentioning the amount of voxels it consists of at uncorrected thresholds. All statistical analyses were repeated with gyrification as regressand at same thresholds and the omission of TIV as a covariate in the multiple regression.

## Results

### ZTPI

After data acquisition we calculated the different time perspective scores for each participant according to the manual and Cronbach’s ⍺ for each scale (Table 1; Funke et al. 2009; Zimbardo and Boyd 1999). None of the factors of the ZTPI were significantly correlated with age or sex, but some of the ZTPI subscales were significantly intercorrelated (Table 1).

**Table 1:** Means, standard deviations, and correlations of ZTPI factors, age and sex

Variable	M	SD	⍺	PN	PH	F	PP	PF	Age
Past negative	2.53	0.65	0.79						
Present hedonistic	3.22	0.45	0.82	0.1					
Future	3.50	0.50	0.78	0.05	-0.21				
Past positive	3.45	0.49	0.84	-0.14	0.18	0.2			
Present fatalistic	2.29	0.52	0.89	0.51*	0.25*	-0.15	-0.09		
Age	29.86	8.93	-	-0.01	-0.15	0.10	-0.08	-0.01	
Sex	0.53	0.50	-	0.18	0.09	0.14	0.19	0.10	0.11

Note. M, SD and ⍺ are used to represent mean, standard deviation and Cronbach’s ⍺, respectively. In columns, ZTPI factors were abbreviated: Past negative (PN), Present hedonistic (PH), Future (F), Past positive (PP), Present fatalistic (PF). Bonferroni-Holm method was employed to correct for multiple hypothesis testing. *indicates *p* < 0.05 after the adjustment procedure. Depicted associations with age and intercorrelations of the ZTPI factors are Pearson correlation coefficients, associations with sex are shown as correlation ratios η

### VBM results

We identified several significant associations between ZTPI factors and grey matter variations in healthy subjects. An overview of these association results with co-ordinates of findings and corresponding anatomical regions is given in Table 2. For display purposes, images are shown with *p* < 0.001 (uncorrected) thresholds, while the table indicates each cluster that met *p* < 0.05 FWE cluster-level correction for multiple comparisons.

**Table 2:** Associations between grey matter volumes and ZTPI factors

Association with ZTPI factors	Co-ordinates of maximum voxel	Anatomical region	*k*	*t*	*p*
Past negative negative association	33; 15; 15	Right anterior insula (50%); right frontal operculum (35%); right central operculum (8%); right inferior frontal orbital gyrus (5%); right cerebral white matter (2%)	1692	4.42	0.036
Future negative association	-2; -70; 56	Right precuneus (45%); left superior parietal lobule (28%); left precuneus (26%)	1685	4.19	0.036
Present fatalistic negative association	33; 28; 12	Right frontal operculum (47%); right anterior insula (40%); right inferior frontal orbital gyrus (8%), right central operculum (3%); right inferior frontal orbital gyrus (3%)	1615	5.06	0.041

Note*.* p at cluster-level. FWE-correction for multiple testing was carried out at α = 0.05. k represents the number of voxels the corresponding cluster consists of at uncorrected thresholds. Atlas labelling was conducted with the neuromorphometrics atlas

**Fig. 1:**
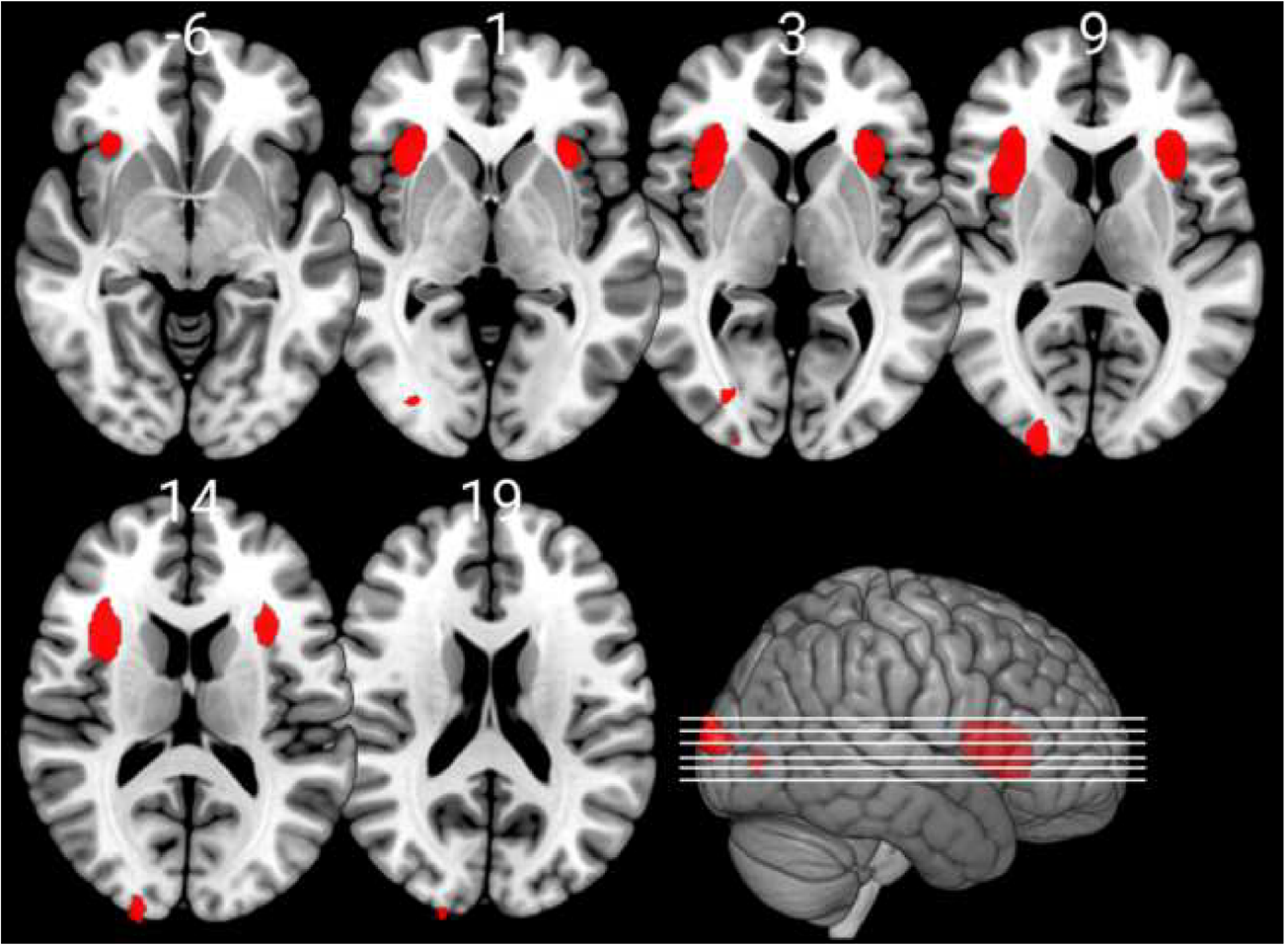
The factor past negative is significantly negatively associated with grey matter volume in the insular cortex and the frontal operculum (*p* < 0.05 FWE corrected at cluster level); note that the images are shown at *p* < 0.001 uncorrected threshold for display purposes

**Fig. 2:**
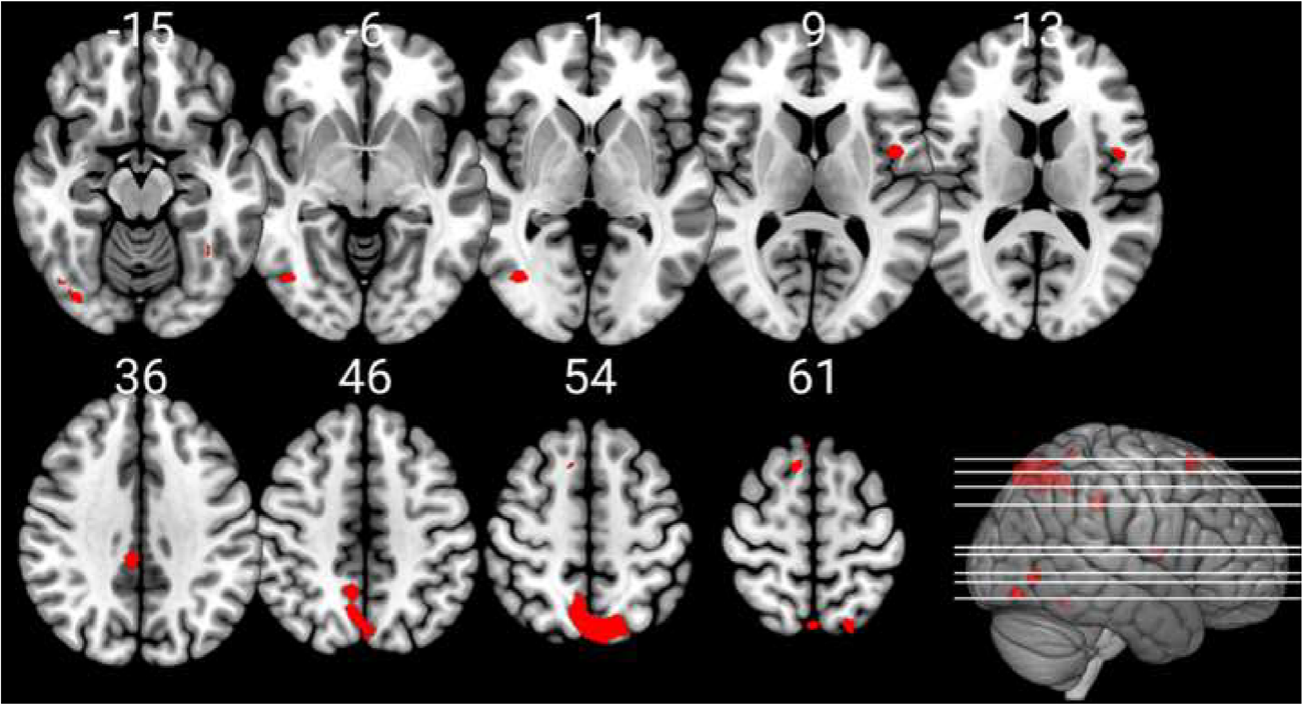
The factor future is significantly negatively associated with grey matter volume in the bilateral precuneus and left superior parietal lobe (*p* < 0.05 FWE corrected at cluster-level); note that the images are shown at *p* < 0.001 uncorrected threshold for display purposes

The past negative scale showed a significant negative association with right anterior insula grey matter, whereas no positive association survived correction for multiple comparisons (Fig. 1). The present hedonistic scale did not show either positive or negative associations with grey matter at the aforementioned thresholds. The future scale showed a significant negative association with a bilateral (mostly right) precuneus cluster, which survived cluster-level correction, but no positive association (Fig. 2). The past positive scale also did not show significant associations with grey matter, but there was a trend (k = 1252, *t* = 4.28, *p* = 0.078, FWE corrected) for a positive association with a right superior/middle temporal cortex cluster. Finally, the present fatalistic scale showed no positive, but a significant negative association with the right frontal operculum and right anterior insula when applying FWE-correction and with the left frontal operculum and anterior insula at trend-level (*k* = 1455, *t* = 4.27, *p* = 0.054, FWE corrected).

### Gyrification results

Neither the positive nor the negative associations between ZTPI factors and gyrification failed to reach significance in the conducted multiple regressions with gyrification data when correcting for multiple testing with FWE-correction.

## Discussion

In this study, we tested the hypothesis that interindividual variation in human time perspective, as assessed psychometrically using the Zimbardo Time Perspective Inventory (ZTPI) is related to variation in brain structure – thus providing a link to the neurobiology of human time perspective. We found associations of ZTPI factors with grey matter variation in several brain areas, mostly notable in the insula and precuneus.

Our results support current theories of the neurobiological basis of human‘s ability to perceive time that have repeatedly understood the insula as playing a main role for this cognitive function. Craig (2009) describes a model that posits that neural substrates of perceiving and estimating time intervals in the range of seconds are located in the anterior insular cortex and proposed a relationship with its interoceptive afferent inputs (Critchley et al. 2004).

Time encoding and reproduction tasks are associated with neural accumulative activation in the medial frontal and insular cortex during the encoding phase and more anterior parts of these brain structures during the reproduction phase (Wittmann et al. 2010). Another study that used a similar design found fMRI activation in the right posterior insula during the encoding phase and activations in the inferior frontal and medial frontal cortices and also in the anterior insula during the reproduction phase (Wittmann et al. 2011). The accumulator-type of the measured neural activity has been interpreted as a unified meta-representation of homeostatic feelings that constitute the conscious self at a time. Through a chain of these accumulation patterns an emotional moment and a highly subjective experience of duration is created. Our findings therefore are consistent with the notion that the insula is involved in meta-representations of time, as shown in the significant negative association between right anterior insula grey matter and the past negative factor and also the significant negative association between right anterior insula grey matter and the scale present fatalistic.

In a delay discounting task, the factor past negative was correlated with impulsivity (Baumann and Odum 2012). The above mentioned accumulative activation of brain regions during reproduction phases of timing phases were also positively correlated with impulsivity (Wittmann et al. 2011), which demonstrates a close relationship between time perspective, impulsivity and time perception of shorter time intervals.

Various cognitive and neural models of time perception have already been proposed. The results of this study and others (Carelli and Olsson 2015; Chen et al. 2018) show striking different associations in very different brain areas. Time perspectives seem to underlay both unity as well as diversity of neural mechanisms. This could be understood as empirical support for intrinsic models of time perception that „assume that sensory and cognitive processes that are not specifically dedicated to time additionally act as interval timers“ in contrast to dedicated models where it is „assumed that the proposed mechanism is exclusively dedicated to measuring duration“ (Wittmann 2013) because there is no specific anatomical brain region which is responsible for time perspective.

The insula finding provides also a link to altered time perspectives in psychopathology. In a meta-analysis, grey matter alterations in the insula have also been identified as a common neurobiological substrate for many mental illnesses, such as schizophrenia, bipolar disorder, depression, anxiety and obsessive-compulsive disorder (Goodkind et al. 2015). Several of these disorders are associated with altered time perspective and perception (Christov-Moore et al. 2014; Gruber et al. 2012; McKay et al. 2016; Moore et al. 2005; Oyanadel and Buela-Casal 2014; Thönes and Oberfeld 2015). A study found that balanced time perspective mediates the relationship between temperament and the severity of PTSD syndrome (Stolarski and Cyniak-Cieciura 2016), a disorder that has also repeatedly been associated with grey matter volumes alterations in the insula (Bromis et al. 2018; Meng et al. 2014).

The factors past negative and present fatalistic are both significantly intercorrelated not only in our sample (Table 1) but also in many other studies (Chen et al. 2018; Gao 2011; Milfont et al. 2008; Wang et al. 2015; Wittmann et al. 2015; Zimbardo and Boyd 1999). This suggests that these scales could potentially measure parts of the two constructs which are shared by both of them and this common intersection is on a neurobiological level correlated with the frontal operculum and the anterior insula. Depression is correlated with past negative and present fatalistic time perspective (Anagnostopoulos and Griva 2012; Wang et al. 2015; Zimbardo and Boyd 1999) as well as with volume reductions in the insula (Kong et al. 2014; Lai 2013; Opel et al. 2015), which links time perspective, brain morphological changes and depression to each other.

Our second main finding is the link between the future time perspective scale and a bilateral precuneus cluster. On the behavioral level, future time perspective is negatively correlated with both loneliness and depressive symptoms (Bergman and Segel-Karpas 2018), and positively with health behavior and well-being (Drake et al. 2008; Kooij et al. 2018). On a structural neural level, a positive relationship has been found between subjective happiness scores and purpose in life scores on the one hand and grey matter volume in the right precuneus on the other (Sato et al. 2015). Taken together, this suggests that there is a positive association between future time perspective and grey matter volume in the precuneus that is moderated by happiness/well-being. However, in this study we demonstrate a negative association between future time perspective and grey matter volume in the precuneus. This could mean that the negative association between future time perspective and grey matter in the precuneus is so strong that it conceals the positive association between these two variables that is moderated by happiness/well-being. However, we did not measure subjective happiness, so that we cannot test its potential moderator effect. This could be an interesting subject for future studies.

Our finding also provides a putative link to episodic memory retrieval, one of the multiple behavioral functions of the precuneus (Cavanna and Trimble 2006; Lundstrom et al. 2005; Lundstrom et al. 2003). According to the constructive episodic simulation hypothesis, there is an overlap between the same cognitive and neural processes that are affected during imagining future events and remembering past events (Schacter et al. 2007; Squire et al. 2010). The theory further posits that imagining the future is pulling apart our recollections and then piecing them together in a montage that might represent a new scenario. Thus, the negative association between grey matter volume in the precuneus and future time perspective supplements existing research that found that autobiographical memory and mental time traveling are cognitive processes that are associated with the same areas of the brain (Addis et al. 2007; Szpunar et al. 2007).

There are only very few directly comparable studies of the neural correlates of time perspective. A recent VBM study that also used the ZTPI (Chen et al. 2018) found a positive association between the factor past negative and grey matter volume in the left prefrontal cortex, a negative association between the factor past positive and grey matter volume in the orbitofrontal cortex and another negative association between future time perspective and the medial prefrontal cortex. The present hedonic scale was positively associated with grey matter in the middle temporal gyrus and present fatalistic time perspective with increases in grey matter in the precuneus. Several factors might account for the differences in findings compared to our study. The two samples differ regarding the age of the subjects and studies showed that the factors present hedonistic and past negative are negatively related to ageing (Laureiro-Martinez et al. 2017). Sex has also a significant effect on time perspective (Diaz-Morales 2006) and distributions of this parameter was also different in the two samples (although accounted for this possible effect in our statistical model). An inspection of the ZTPI scale also demonstrated some correlations with personality traits (Adams and Nettle 2009; Muro et al. 2015) what could also account for the differences in the studies.

Cultural background might also modulate both the construct of time perception as well as its neurobiological basis since time is a social institution (Sircova et al. 2015). Other factors that could have modulated the found associations are perceived stress, impulsivity, well-being and resilience (Baumann and Odum 2012; Garcia et al. 2016; Rönnlund et al. 2018; Tseferidi et al. 2017; Wittmann et al. 2011).

This new study uses both VBM aiming to replicate a previous finding on ZTPI, as well as gyrification, a more novel marker for subtle early brain developmental effects, in order to provide a multi-modal approach to brain structure and time perspective taking in humans. It provides novel findings for our understanding of this complex trait. Our lack of findings for gyrification suggests that the identified brain structural substrates of time perspective are not related to subtle variation in early brain development. Gyrification reflects a basic folding property of the (neo)cortex. Once basic brain development is completed in early childhood, this marker is thought to remain rather stable across most of adulthood. Unlike VBM, which might be susceptible to state-related changes or fluctuations of regional brain volumes, gyrification is thus expected to reflect the intrinsic geometry of cortical folds.

We need to consider some methodical limitation. First, we did not survey some variables that would allow us to use more complex statistical models, e.g. moderator- and mediator analyses. As a result we would be able to integrate well-being, time perception and psychopathology directly in our data. Second, some effects did not survive correction for multiple testing but might still be relevant. Future studies with higher statistical power could potentially capture some effects we could not detect.

In conclusion, our study shows an association of time perspective with grey matter variation in the anterior insula and precuneus. This links time perspective to other aspects of processing of temporal information and provides links to altered time perspective in psychopathology as well as links to autobiographical memory.
